# A rare association of crossed fused renal ectopia

**DOI:** 10.1186/1471-2369-8-5

**Published:** 2007-03-01

**Authors:** Riaz Ahmad

**Affiliations:** 1 Department of Orthopaedics, Weston General Hospital, Weston-Super-Mare, UK

## Abstract

**Background:**

Thrombocytopenia and absent radius syndrome (TAR) is a rare genetic disorder. It is an autosomal recessive disorder characterised by radial aplasia and thrombocytopenia that may have additional anomalies. We report a case of TAR syndrome with crossed fused renal ectopia. This anomaly has not been previously reported in association with TAR syndrome.

**Case presentation:**

A 24 years old female with Thrombocytopenia and absent radius syndrome admitted with pelvic fracture was investigated for recurrent urinary tract infections. Abdominal ultrasonography could not visualise the kidney on right side. Further extensive investigations in the form of intravenous urography (IVU), Magnetic resonance imaging (MRI) and renal isotope scans revealed a crossed fused renal ectopia.

**Conclusion:**

This report describes the new finding of a crossed fused renal ectopia associated with TAR syndrome that has not been reported before in the literature. Ectopic kidneys have increased susceptibility to develop complications like urinary infections, urolithiasis, and abdominal mass. There is a reported case of TAR syndrome with renal anomaly that developed Wilm's tumor. Finding of crossed fused renal ectopia warrants complete urologic investigation to rule out surgically correctable pathology in the urinary tract.

## Background

**Figure 1:**
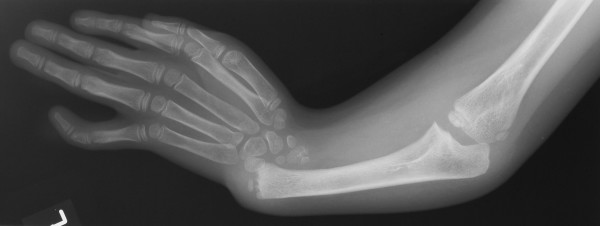
AP radiograph of the upper limb showing absent radius with radially deviated hand.

TAR syndrome is an autosomal recessive disorder with constant findings of thrombocytopenia and bilateral absence of radii with presence of thumbs (Figure 1). Many of the congenital anomalies have been described such as ulnar hypoplasia, malformed humeri, leucocytosis, tetralogy of fallot, atrial septal defect, ventricular septal defect and milk protein allergy [1].There are only three reports of renal anomalies associated with TAR syndrome.

Bradshaw et al. [2] reported a patient with TAR syndrome and horse shoe kidney, Chappel [3] reported TAR syndrome with penoscrotal transposition, i.e., insertion of penis below scrotum , Fivush et al. [4] reported TAR syndrome with bilateral hypoplastic kidneys and poor renal function. Crossed fused renal ectopia is a very rare anomaly in which both kidneys are located on the same side and are fused. The autopsy incidence of renal ectopia is 5.9%.

We report the first patient of TAR syndrome associated with crossed fused renal ectopia and discuss the pathogenetic explanation for crossed fused renal ectopia.

## Case presentation

**Figure 2:**
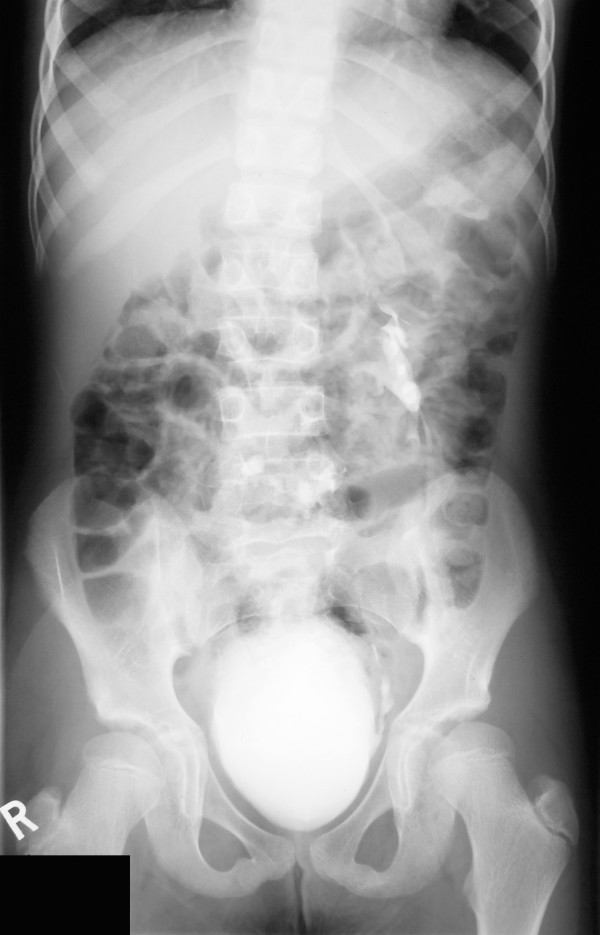
Intravenous Urogram showing crossed fused renal ectopia.

**Figure 3:**
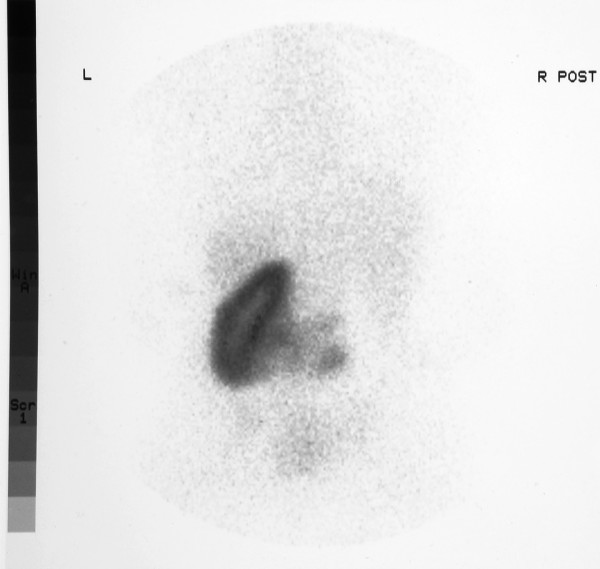
Isotope scan showing an ectopic kidney on the left side.

**Figure 4:**
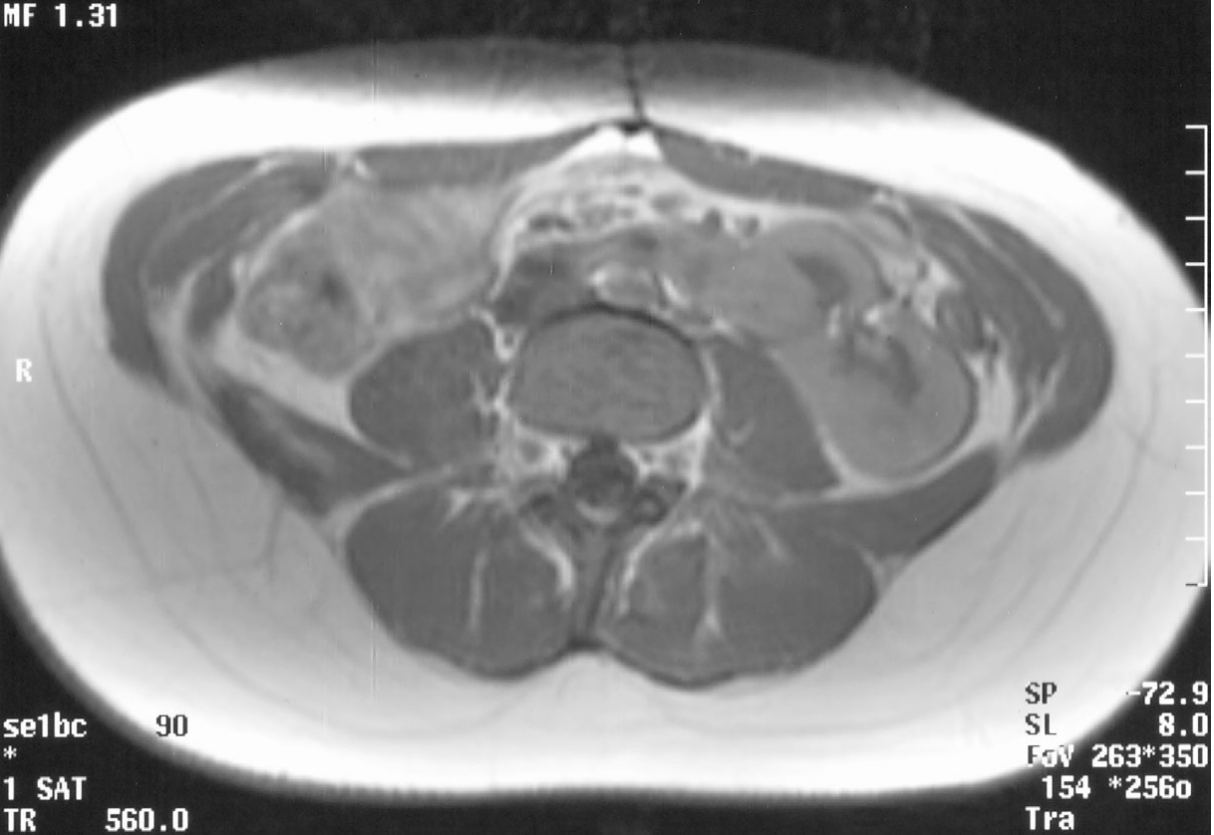
MRI Scan showing a crossed fused kidney on the left side.

A 24 years old female with Thrombocytopenia absent radius syndrome (TAR) was admitted with fracture of pelvis in our department. Diagnosis of TAR syndrome had been made on the basis of radiographic findings of absent radii, radially deviated hands, presence of thumbs and a low platelet count. There was no family history of consanguinity or congenital malformations. USG was performed for recurrent urinary tract infections which revealed absent kidney on the right side. Further investigations including IVU, MRI scan and renal isotope scans revealed a crossed fused renal ectopia (fig 2, 3, 4).

## Conclusion

TAR syndrome was first described by Hauser in 1948. In 1956 Gross et al. [5] described it as a group of limb abnormalities including absent radii, ulnar hypoplasia and malformed humeri with hypo megakaryocytic thrombocytopenia. Genitourinary anomalies have been described in three cases of Tar Syndrome [[1,3] and [4]]. One infant had bilateral hypoplastic kidneys with poor renal function. The second child had transposition of external genitalia. The third child had TAR syndrome with a horse shoe kidney. Our patient represents fourth such case but with crossed fused renal ectopia.

Renal fusion anomalies can be categorized into 2 varieties: (1) horseshoe kidney and its variants and (2) crossed fused ectopia. Horseshoe kidney is probably the most common fusion anomaly. The term horseshoe kidney refers to the appearance of the fused kidney, which results from fusion at one pole. Horseshoe kidney is differentiated from crossed fused ectopia in which both fused kidneys lie on one side of the spine and the ureter of the crossed kidney crosses the midline to enter the bladder.

Crossed fused renal ectopia is a rare renal anomaly with incidence of 1:1300 – 1: 7500 [6]. The formation of metanephros – the developing kidney depends on the presence of both the ureteric bud and metanephric blastema. The ureteric bud arises from the lower portion of wolffian duct and the metanephric blastema is a mesoderm tissue. Both these tissues migrate towards each other and merge to form the kidney and the urinary tract. Over bending and rotation of the caudal end of the embryo prevents the ureteric bud from merging with the ipsilateral metanephric blastema and thus is attracted towards the now more closer contralateral side[7].There is an increased prevalence of crossed renal ectopia in patients with scoliosis which supports the above theory [8].

With growth the kidneys gradually ascend to be in the abdomen and away from the midline. Since the under ascent is more common than the over ascent, the ectopic kidneys are more commonly found in the pelvis or the lower abdomen. In most cases the fusion is between the lower pole of the orthotopic kidney and the upper pole of the ectopic kidney, it is usually the left side kidney which crosses to the right. In a recent clinical study of 34 patients with TAR syndrome Greenhalgh et al. found that renal anomalies occurred in seven cases (23%) [9].

Most of the renal anomalies are incidental findings and the ectopic kidneys have a high incidence of stone formation. Although most of the patients with crossed fused renal ectopia are usually asymptomatic, they do present with increased susceptibility to develop complications like urinary infections, urolithiasis, and abdominal mass. There are reported cases of renal cell carcinoma and Wilm's tumor associated with crossed fused renal ectopia [10,11]. There is a reported case of TAR syndrome with renal anomaly that developed Wilm's tumor. [9] Finding of crossed fused renal ectopia warrants complete urologic investigation to rule out surgically correctable pathology in the urinary tract.

In summary, this report describes the new finding of a crossed fused renal ectopia associated with TAR syndrome. This association has not been reported before in the literature.

## Competing interests

The author(s) declare that they have no competing interests.

## Pre-publication history

The pre-publication history for this paper can be accessed here:


